# AMP-Activated Protein Kinase Interacts with the Peroxisome Proliferator-Activated Receptor Delta to Induce Genes Affecting Fatty Acid Oxidation in Human Macrophages

**DOI:** 10.1371/journal.pone.0130893

**Published:** 2015-06-22

**Authors:** Marina Kemmerer, Florian Finkernagel, Marcela Frota Cavalcante, Dulcineia Saes Parra Abdalla, Rolf Müller, Bernhard Brüne, Dmitry Namgaladze

**Affiliations:** 1 Institute of Biochemistry I, Faculty of Medicine, Goethe-University Frankfurt, Theodor-Stern-Kai 7, 60590, Frankfurt, Germany; 2 Institute of Molecular Biology and Tumor Research (IMT), Philipps-University Marburg, Marburg, Germany; 3 Department of Clinical and Toxicological Analyses, Faculty of Pharmaceutical Sciences, University of Sao Paulo, Sao Paulo, Brazil; INRA, FRANCE

## Abstract

AMP-activated protein kinase (AMPK) maintains energy homeostasis by suppressing cellular ATP-consuming processes and activating catabolic, ATP-producing pathways such as fatty acid oxidation (FAO). The transcription factor peroxisome proliferator-activated receptor δ (PPARδ) also affects fatty acid metabolism, stimulating the expression of genes involved in FAO. To question the interplay of AMPK and PPARδ in human macrophages we transduced primary human macrophages with lentiviral particles encoding for the constitutively active AMPKα1 catalytic subunit, followed by microarray expression analysis after treatment with the PPARδ agonist GW501516. Microarray analysis showed that co-activation of AMPK and PPARδ increased expression of FAO genes, which were validated by quantitative PCR. Induction of these FAO-associated genes was also observed upon infecting macrophages with an adenovirus coding for AMPKγ1 regulatory subunit carrying an activating R70Q mutation. The pharmacological AMPK activator A-769662 increased expression of several FAO genes in a PPARδ- and AMPK-dependent manner. Although GW501516 significantly increased FAO and reduced the triglyceride amount in very low density lipoproteins (VLDL)-loaded foam cells, AMPK activation failed to potentiate this effect, suggesting that increased expression of fatty acid catabolic genes alone may be not sufficient to prevent macrophage lipid overload.

## Introduction

The number of people with diabetes is expected to rise to 366 million in 2030 worldwide [1]. Patients with the metabolic syndrome—symptoms of which include abdominal obesity, dyslipidemia, glucose intolerance, and hypertension—have a five-fold increased risk of developing type 2 diabetes mellitus and usually show a decreased capacity for exercise [2–6]. The connection between metabolism and immune responses is increasingly being appreciated in the context of metabolic diseases, including atherosclerosis and obesity-driven diabetes [7, 8]. Particularly, lipid metabolism in macrophages undergoing foam cell formation is crucial to regulate inflammatory processes in developing atherosclerotic plaques and expanding adipose tissue [9, 10]. During foam cell formation macrophages take up considerable amounts of lipids and adapt to lipid loading by activating transcriptional programmes aimed at preventing excessive lipid overload and limiting inflammation.

Transcription factors of the peroxisome proliferator-activated receptor (PPAR) family (PPARα, -δ and -γ) are critical for adaptation to lipid overload [11]. PPARδ acts as a heterodimer with the retinoid X receptor (RXR), binding to PPAR response element (PPRE) DNA sequences [12]. There are three different types of target gene regulation by PPARδ: agonist-independent repression (type I); agonist-sensitive repression (type II), and agonist-independent activation (type III) [13]. In case of type II regulation, PPARδ induces a repressive state by executing a transcriptional co-repressor function in the absence of agonists. Once activated by a ligand, the heterodimer PPARδ-RXR recruits co-activators promoting initiation of gene transcription [14]. Among the different PPARs PPARδ is most ubiquitously expressed and may be particularly relevant for macrophages handling triglyceride-rich lipoproteins [15]. Recently, we and others found that PPARδ is activated in triglyceride-rich foam cells following the uptake of phospholipolyzed lipoproteins or very low density lipoproteins (VLDL). Subsequently, activated PPARδ attenuates inflammatory responses in macrophages [16, 17]. This is consistent with the anti-atherogenic effects of PPARδ in animal models [18–20]. Transcriptional reprogramming of macrophage lipid metabolism by PPARδ is primarily characterized by increased mitochondrial and peroxisomal fatty acid oxidation (FAO) [21, 22], similar to the effects of PPARδ activation in metabolically active tissues such as skeletal muscle [23]. Induction of FAO was linked to anti-obesity and insulin-sensitizing *in vivo* phenotypes following PPARδ activation [22, 23].

In addition to transcriptional regulators, AMP-activated protein kinase (AMPK) plays a key role to connect metabolism and inflammation [8]. AMPK senses metabolic stresses via its activation by increased AMP/ATP and ADP/ATP ratios. Activated AMPK shuts off energy-consuming processes, while inducing protein, carbohydrate, and fat catabolism. AMPK activates FAO through phosphorylation and inactivation of acetyl-CoA carboxylase (ACC) thus, reducing levels of malonyl-CoA, an allosteric inhibitor of carnitine palmitoyltransferase (CPT1a) [8]. AMPK also inactivates glycerol-3-phosphate acyltransferase, channeling acyl-CoA towards β-oxidation [24]. This may underlie insulin-sensitizing effects of AMPK activation, and contribute to anti-inflammatory functions of AMPK in adipose tissue macrophages [25]. An allosteric AMPK activator A-769662 has been described to act independently of the upstream AMPK kinases, inhibiting AMPK dephosphorylation [26] and to decrease plasma glucose and triglyceride levels in a mouse diabetes model ob/ob mice [27].

As both, AMPK and PPARδ provide beneficial metabolic effects, at least in part by targeting FAO, it is of interest how these regulators cooperate. Previous studies in skeletal muscle explored the interaction of AMPK and PPARδ and showed that combined pharmacological activation of AMPK and PPARδ in mice created an unique phenotype associated with increased running endurance through enhanced muscle fatty acid metabolism [28]. It was proposed that the combined activation of AMPK and PPARδ may provide additional metabolic benefits compared to single treatments. In our study we explored the transcriptome of human macrophages under conditions of single and combined activation of AMPK and PPARδ. We found enhanced activation of FAO-associated genes by combined AMPK/PPARδ agonism. However, these transcriptional changes were not accompanied by enhanced macrophage FAO or protection against lipid overload.

## Materials and Methods

### Cell Culture

Human peripheral blood monocytes were isolated from buffy coats provided by anonymous donors (DRK-Blutspendedienst Baden-Württemberg-Hessen, Institut für Transfusionsmedizin und Immunhämatologie, Frankfurt, Germany, URL:http://www.blutspende.de/en/institutes-affiliates/frankfurt-am-main/frankfurt-am-main.php) using Ficoll gradient (LSM 1077, GE Healthcare) centrifugation according to the manufacturer’s protocol and CD14 microbead selection (Miltenyi Biotec). Monocytes were seeded for differentiation in serum-free medium (Macrophage-SFM, Life Technologies), supplemented with 50 ng/ml human recombinant macrophage colony-stimulating factor (M-CSF, Immunotools) and maintained for 6 days. For treatments, cells were incubated in RPMI 1640 medium (GE Healthcare) supplemented with 10% fetal calf serum (FCS), 2 mM glutamine, 100 U/ml penicillin, and 100 μg/ml streptomycin. Stimulation with GW501516 (Axxora), salicylate (Sigma-Aldrich) and A-769662 (LC Laboratories) was for 24 or 48 hours. This investigation conforms to the ethical principles outlined in the Declaration of Helsinki and was approved by the university ethics committee (Ethik-Kommission des Fachbereichs Medizin der Goethe-Universität Frankfurt am Main). The ethics committee waived the need for consent when using the blood of anonymous donors.

THP-1 human acute monocytic leukemia cells (ATCC) were maintained in RPMI 1640 medium, supplemented with FCS, glutamine, penicillin, and streptomycin.

### Plasmid constructs

Cloning of the human truncated AMPKα1 subunit was performed using SBI System Biosciences, Clone-it Enzyme free Lentivectors according to the manufacturer’s protocol. Lentivector LF521A-1 containing a puromycin resistance was used. Briefly, isolated total DNA of human primary macrophages served as a template in PCR using High Fidelity DNA Polymerase (Roche). The following primers were used: AMPK1-for– 5’-atgcgcagactcagttcctg-3’; AMPK2-rev– 5’-ggcaactgccaaaggatcc-3’; AMPK2-for– 5’-GAGGCAGCAGAGACCGatgcgcagactcagttcctg-3’; AMPK1-rev– 5’-CGAACAGAGAGAGA-CCGggcaactgccaaaggatcc-3’. PCR products were cleaned by QIAquick PCR Purification Kit (Qiagen) and annealed by heating the mix at 95°C and slowly cooling down. Following transformation resulting clones were verified by sequencing. For lentiviral production the lentiviral packaging vector pCMV-dR8 and the viral envelope plasmid pMD2G were used. Adenoviruses coding for AMPKγ1 regulatory subunit carrying an activating R70Q substitution were kindly provided by Dr Jason Dyck, Cardiovascular Research Centre, University of Alberta, Canada. Control adenoviruses (AdTrack) were from Addgene.

### Site-directed mutagenesis

Threonine-172 to aspartic acid mutation (T172A) of AMPKα1 was performed by QuickChange II XL Site-directed Mutagenesis Kit (Agilent Technologies) using PfuUltra HF DNA polymerase (Stratagene). Following mutagenesis primers were used: mutAMPK-for—5’-cagatggtgaatttttaagagatagttgtggctcacccaactatgc-3’; mutAMPK-rev– 5’-gcatagttgggtgagcc-acaactatctcttaaaaattcaccatctg-3‘. The inserts were sequenced in their entirety in order to confirm the authenticity of the mutation and to ensure no other mutations occurred. Mutation creates a constitutively active AMPKα1 subunit consisting of 312 amino acids. This truncated form of AMPKα1 was used in the microarray analysis.

### Lentiviral production

2.5*10^6^ 293T HEK cells were seeded in 10 cm dishes and transfected by pCMV-dR8 and pMD2G plasmids (proportion 5.25:1) together with a control vector pCDH-EF1-T2A-puro or LF521A-1-puro-AMPKα1 using JetPRIME transfection reagent (Polyplus) followed by medium exchange 24 hours post-transfection. Supernatants containing lentiviral particles were collected 72 hours post-transfection and concentrated using Lenti-X concentrator (Clontech) according to manufacturer’s protocol.

### Microarray analysis

Primary human macrophages were transduced with control (CV) or lentiviral particles encoding the constitutively active AMPKα1 (AMPK OE) for 48 hours and treated with 100 nM GW501516 for additional 24 hours. Total RNA isolation was performed by phenol-chloroform extraction. RNA was further purified by RNeasy total RNA Cleanup Kit (Qiagen) and eluted in RNase-free water. RNA quality was analyzed by the Agilent RNA 6000 analyzer. Whole genome microarray analysis was performed using the Illumina Sentrix Human HT-12 v4 chip. Raw microarray data were normalized using the VSN method and assigned to human gene symbols using R/Bioconductor [29] and the bead array package [30]. Triplicates were contrasted using Limma [31] and differentially expressed genes were selected based on a 1.5-fold change and a Benjamini-Hochberg adjusted p-value smaller than 0.1. Functional annotation was performed using Gene Set Enrichment Analysis [32] against gene sets derived from the molecular signature database version 3.1, datasets c2, c3 and c6 [32], from Pathway Commons [33] and from Genome Ontology via Ensembl, revision 70 [34].

### RNA extraction, reverse transcription, and real-time quantitative PCR

Total RNA of 1*10^6^ cells was isolated using peqGOLD RNAPure (PeqLab) according to manufacturer´s protocol. 1 μg of total RNA was reverse transcribed using the Maxima First Strand cDNA Synthesis Kit (Thermo Scientific). Real-time quantitative PCR assays were performed with the iQ Custom SYBR Green Supermix (Bio-Rad) using the CFX96 system from Bio-Rad. Each amplification sample contained 20 ng of cDNA, 250 nM each of forward and reverse primers and 5 μl of 2x iQ SYBR Green Supermix. The mRNA expression was normalized to GAPDH. Following primers were used for quantitative PCR: PDK4-forward—5’-cctttggctggttttggtta-3’; PDK4-reverse– 5’-cctgcttgggatacaccagt-3’; CPT1a-forward– 5’-tcgtcacctcttctgccttt-3’; CPT1a-reverse– 5’-acacaccatagccgtcatca-3’; PLIN2-forward– 5’-aagaaaaatggcatccgttg-3’; PLIN2-reverse– 5’-caatttgcggctctagcttc-3’; PPARδ-forward– 5’-tcacacagtggcttctgctc-3’; PPARδ-reverse– 5’-tctacagggtggttcccatc-3’; Angptl4-forward– 5’-gcctatagcctgcagctcac-3’; Angptl4-reverse– 5’-agtactggccgttgaggttg-3’; GAPDH-forward– 5’-tgcaccaccaactgcttagc-3’; GAPDH-reverse– 5’-ggcatggactgtggtcatgag-3’.

### Western analysis

Cell pellets were harvested in lysis buffer (50 mM Tris-HCl pH 8, 150 mM NaCl, 5 mM EDTA, 10 mM NaF, 1 mM Na_2_VO_4_, 0.5% NP-40, 1 mM PMSF, protease inhibitor cocktail (Roche)). Nuclei were isolated using nuclear lysis buffer A (20 mM Tris-HCl pH 8.0, 10 mM NaCl, 5 mM EDTA, 0.5% NP-40, 1 mM PMSF, protease inhibitor cocktail) followed by centrifugation at 16000g for 20s. Nuclear pellets were sonicated in lysis buffer B (20 mM Tris-HCl pH 8.0, 400 mM NaCl, 5 mM EDTA, 0.5% NP-40, 1 mM PMSF, protease inhibitor cocktail). 50–100 μg of protein extracts were separated using 8% polyacrylamide gels, transferred to nitrocellulose membrane, blocked in 5% nonfat milk in TBS-Tween buffer (0.1% Tween 20, pH 7.4) and incubated with a desired primary antibody overnight at 4°C. Primary antibodies directed against PDK4 (Abcam, ab38242 and Proteintech Europe, 12949-1-AP), CPT1a (Proteintech Europe, 15184-1-AP), phospho-AMPK (#2531), AMPKα (#2532), AMPKβ (#4150), phospho-ACC (#3661), ACC (#8578), phospho-S6 (#4856), S6 (#2317) (all Cell Signaling Technology), PPARδ (Santa Cruz Biotechnology, sc-74517 and Abcam, ab58137) and nucleolin (Santa Cruz Biotechnology, sc-13057) were used followed by IRDye 680 or IRDye 800-coupled secondary antibodies (LICOR Biosciences). Nucleolin was used for normalizing the amount of protein loaded onto each lane.

### Oxygen consumption measurements

Macrophages were plated in Seahorse cell culture plates and treated as indicated. For oxygen consumption measurements the medium was changed to Krebs-Henseleit buffer (111 mM NaCl, 4.7 mM KCl, 1.25 mM CaCl2, 2 mM MgSO4, 1.2 mM NaH2PO4) supplemented with 0.5 mM carnitine, 5 mM HEPES and 100 μM palmitate-BSA conjugate, adjusted to pH 7.4 at 37°C, prior to the assay. FAO was measured using Seahorse 96 extracellular flux analyzer (Seahorse Bioscience) as the difference in oxygen consumption rates before and after the addition of 25 μM CPT1a inhibitor etomoxir and was normalized to the protein amounts in the wells.

### Triglyceride measurement

Human VLDL was isolated from the plasma samples of healthy volunteers by sequential ultracentrifugation. Primary macrophages were pretreated with 100 nM GW501516 and/or 250 μM A-769662 for 48 hours. After medium changes cells were loaded by 20 μg/ml VLDL for additional 24 hours. Triglyceride (TG) content was determined using TG determination kit (Roche) according to the manufacturer’s instructions and normalized to protein content.

### siRNA-mediated RNA interference

siRNAs (ON-Target plus SMARTpool, Dharmacon) targeting human PPARδ, AMPKα1 or scrambled control RNA oligonucleotides were transfected into primary macrophages at a final concentration of 50 nM for 72 hours using Hiperfect transfection reagent (Qiagen) according to the manufacturer’s instructions. Stimulation with 250 μM A-769662 or 100 nM GW501516 for additional 24 hours followed.

### Statistical analysis

The significance of the differences in mean values among two groups was evaluated by one-way ANOVA test. Differences were considered statistically significant for p<0.05 (*/#/§) and p<0.01 (**/##). Data are presented as averages ± 95% Confidence Interval of at least three independent experiments.

## Results

**Fig 1:**
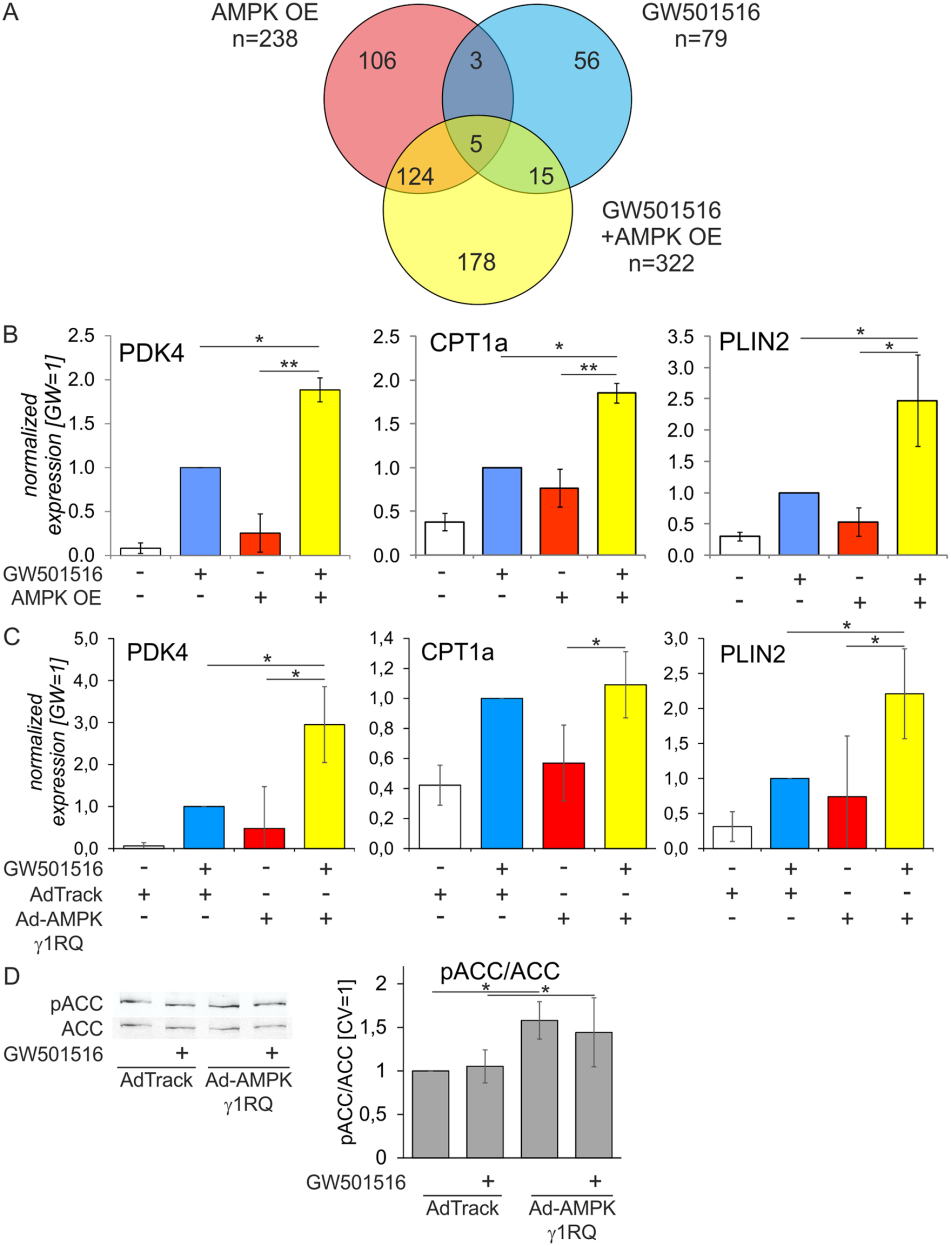
Analysis of AMPK and PPARδ interactions in primary human macrophages with overexpression of AMPK catalytic or regulatory subunits. Primary human macrophages were transduced with control lentivirus (CV) or lentiviral particles coding for a constitutively active AMPKα1 (AMPK OE) for 48 hours and treated with 100 nM GW501516 for additional 24 hours. **A** Venn diagram showing numbers of genes regulated by AMPK OE, GW501516, or their combination. **B** Validation of microarray analysis by quantitative PCR. **C** mRNA expression of PDK4, CPT1a, and PLIN2 in macrophages infected with control adenovirus (AdTrack) or AMPKγ1 R70Q adenovirus for 48 hours prior to 24 hour-treatment with 100 nM GW 501516. **D** Western blot showing ACC phosphorylation and its quantification in macrophages infected with control adenovirus (AdTrack) or AMPKγ1 R70Q adenovirus for 48 hours. Values represent averages ± 95% Confidence Interval. *, p<0.05; **, p<0.01 (n = 3).

Previous studies revealed interactions of AMPK and PPARδ in muscle cells, causing a transcriptional reprogramming to increase fatty acid oxidative metabolism [28]. We questioned functional interactions of AMPK and PPARδ in primary human macrophages by analyzing alterations of the macrophage transcriptome following single and combined treatments to activate AMPK and PPARδ. To avoid off-target effects associated with pharmacological AMPK activation we overexpressed a lentiviral construct coding for a truncated, constitutively active human AMPKα1 subunit (AMPK OE) in primary human macrophages, followed by 24 hour-treatments with 100 nM of the selective PPARδ agonist GW501516. Genome-wide mRNA expression profiling was then performed using Illumina HT12v4 bead arrays (EMBL-EBI Array Express accession number E-MTAB-2524). As illustrated by the Venn diagram (Fig 1A), 238 genes were regulated by AMPK overexpression (107 up, 131 down, log_2_(fold change)≥0.58), while 79 genes changed their expression in response to GW501516 (46 up, 33 down) with an overlap of 8 genes (3 up, 5 down). Combined AMPK/PPARδ activation altered the expression of 322 genes (128 up, 194 down). Testing the cooperativity of gene regulation by AMPK and PPARδ we did not find any probe showing >50% difference in intensity after co-activation of AMPK and PPARδ compared with single stimulations, indicating no synergistic effects.

Gene Set Enrichment Analysis (GSEA) is a method to interpret gene expression data focusing on gene sets [32]. Comparing combined AMPK/PPARδ activation with untreated samples revealed fatty acid oxidative metabolism dominating the list of 20 mostly enriched pathways (Table 1). Among the 20 strongest induced genes upon GW501516-treatment and AMPK OE shown in Table 2, 7 referred to fatty acid metabolism, including FAO-associated genes pyruvate dehydrogenase kinase 4 (PDK4), CPT1a, acetyl-CoA acyltransferase 2 (ACAA2), and very long chain acyl-CoA dehydrogenase (ACADVL). Perilipin 2 (PLIN2), a common PPARδ target gene, was also present in this list.

**Table 1:** Major regulated pathways by Gene Set Enrichment Analysis.

Gene set	Size	NES
Fatty acid beta oxidation	38	2.17
Kinesin binding	20	2.16
ATP biosynthetic process	32	2.15
Pyruvate metabolism and TCA cycle	38	2.13
TCA cycle and respiratory electron transport	110	2.08
Ion transport by P-type ATPases	31	2.07
FFA oxidation	22	2.00
STAT3 targets	46	1.99
TCA cycle	18	1.97
Pyruvate metabolic process	24	1.96
Oxidative stress response	33	1.96
Pyruvate metabolism	17	1.96
Endoplasmic reticulum organization	16	1.95
Cellular aromatic compound metabolic process	7	1.95
PPAR signaling pathway	68	1.93
Hydrolase activity, acting on acid anhydrides, catalyzing transmembrane movement of substances	41	1.91
Estrogen metabolic process	12	1.91
Positive regulation of fatty acid beta oxidation	8	1.90

NES, normalized enrichment score

**Table 2:** 20 strongest induced genes upon combined AMPK/PPARδ activation.

Gene symbol	Name	log_2_ (fold change), GW+AMPK OE vs. CV
PDK4	pyruvate dehydrogenase kinase, isozyme 4	2.11
CPT1A	carnitine palmitoyltransferase 1A (liver)	1.48
FABP4	fatty acid binding protein 4, adipocyte	1.30
PLIN2	perilipin 2	1.08
ACAA2	acetyl-CoA acyltransferase 2	1.03
UCHL1	ubiquitin carboxyl-terminal esterase L1 (ubiquitin thiolesterase)	0.96
RNF128	ring finger protein 128, E3 ubiquitin protein ligase	0.96
DHRS9	dehydrogenase/reductase (SDR family) member 9	0.95
ELOVL6	ELOVL fatty acid elongase 6	0.91
FAM160B1	family with sequence similarity 160, member B1	0.91
IMPA2	inositol (myo)-1 (or 4)-monophosphatase 2	0.87
ANKDD1A	ankyrin repeat and death domain containing 1A	0.84
SPINK1	serine peptidase inhibitor, Kazal type 1	0.83
ACADVL	acyl-CoA dehydrogenase, very long chain	0.82
ZNF366	zinc finger protein 366	0.81
HPSE	heparanase	0.81
SEMA3E	semaphorin 3E	0.79
CDH23	cadherin-related 23	0.79
NBL1	neuroblastoma, suppression of tumorigenicity 1	0.79
CD36	CD36 molecule (thrombospondin receptor)	0.76

We then confirmed that FAO is the major pathway undergoing transcriptional activation after AMPK and PPARδ co-activation. Validation by quantitative PCR analysis showed enhanced induction of FAO-associated genes PDK4 and CPT1a, as well as PLIN2 after a single treatment with GW501516 or after AMPK overexpression (Fig 1B). Besides, we observed a significantly increased PDK4, CPT1a, and PLIN2 mRNA expression in PPARδ/AMPK-coactivated macrophages compared to individual activation.

The truncated AMPK construct used here lacks the interaction with regulatory subunits, which may be important for AMPK substrate targeting. Indeed, we failed to detect increased phosphorylation of the AMPK substrate ACC in macrophages with AMPK OE (data not shown), similar to previous observations in rat cardiomyocytes [35]. Therefore, to validate our observations we infected macrophages with adenoviruses coding for a regulatory AMPKγ1 subunit having a R70Q substitution. This mutation was reported to increase the activity of AMPK heterotrimers [36]. Transduction of macrophages with AMPKγ1 R70Q adenovirus replicated effects of the truncated AMPK OE construct on PPARδ target mRNA expression with the exception of CPT1a (Fig 1C). Fig 1D shows that this construct also caused modest, but significant elevations of ACC phosphorylation.

**Fig 2:**
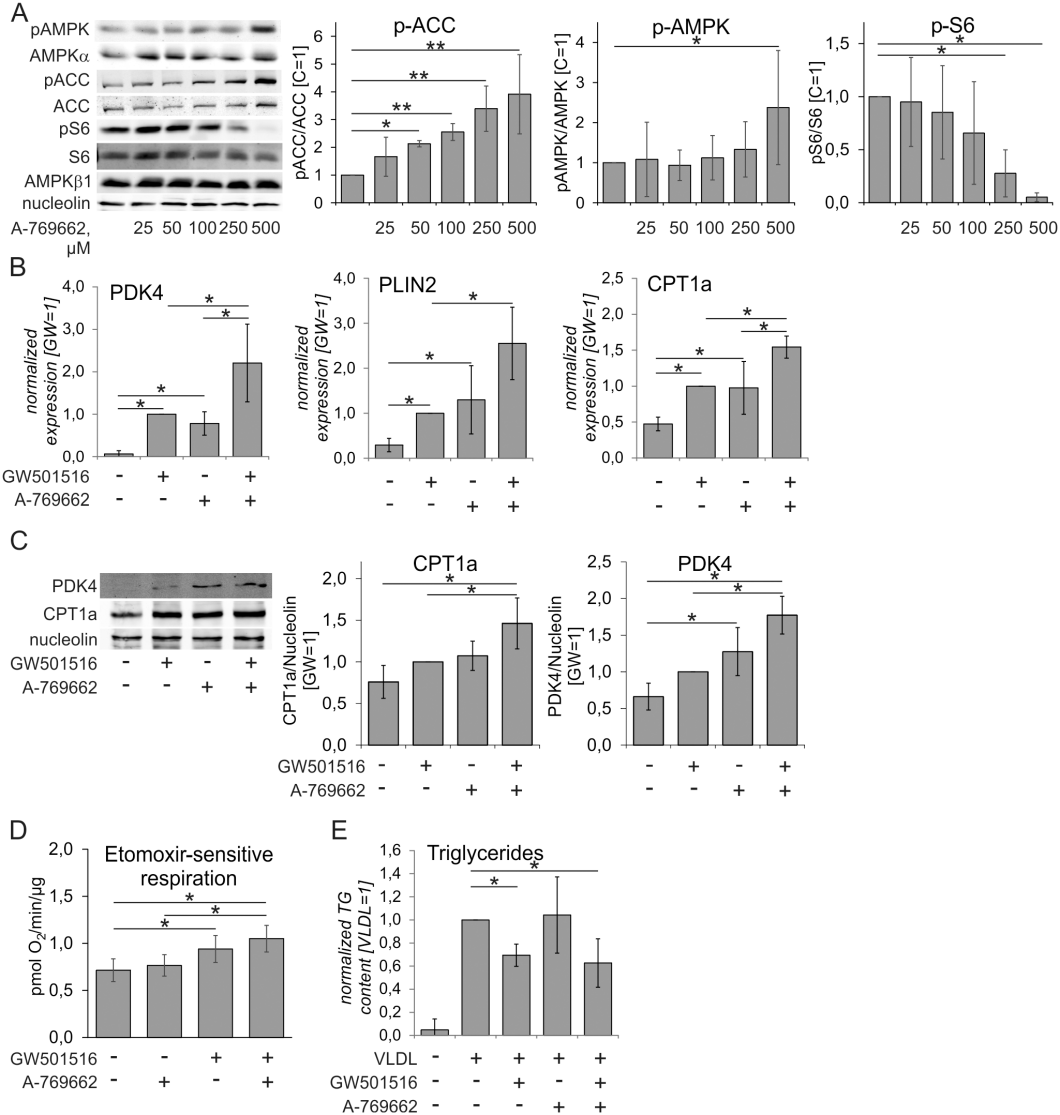
Pharmacological AMPK and PPARδ activation affects expression of FAO genes, FAO and VLDL-induced lipid accumulation in primary human macrophages. **A** Western analysis of macrophages treated with indicated concentrations of A-769662 for 1 hour (n = 4). C, untreated cells. **B**, **C** mRNA (**B**) and protein (**C**) expression of PDK4, CPT1a, and PLIN2 in macrophages treated with 500 μM A-769662 and/or 100 nM GW501516 for 24 hours (mRNA) or 48 hours (protein) (n = 3). **D** Etomoxir-sensitive respiration in human macrophages following 48 hour-treatment with 100 nM GW501516 and 100 μM A-769662. **E** Triglyceride content of primary macrophages treated with 100 nM GW501516 and/or 250 μM A-769662 for 48 hours prior to VLDL (20 μg/ml) stimulation for 24 hours (n = 4). Values represent averages ± 95% Confidence Interval.*, p<0.05; **, p<0.01.

Next, we questioned whether pharmacological AMPK activation similarly enhanced PPARδ target gene mRNA expression as AMPK overexpression. In these experiments we used the allosteric AMPK activator A-769662. Analysis of the phosphorylation status of AMPK, its substrate ACC and ribosomal protein S6, which served as readout of mTOR (mechanistic target of rapamycin) activity, revealed that significant ACC phosphorylation was already observed at 50 μM A-769662, and continued to increase up to 500 μM A-769662 (Fig 2A). In contrast, significant down-regulation of phospho-S6 and up-regulation of phospho-AMPK was achieved only at 250 μM and 500 μM A-769662, respectively. Therefore, we used 500 μM of the drug in subsequent experiments. Measuring intracellular ATP did not show changes after exposure to these concentrations of A-769662, ruling out a loss of viability (data not shown). As shown in Fig 2B, treatment of primary human macrophages with A-769662 induced mRNA expression of the PPARδ target genes PDK4, CPT1a, and PLIN2 to a similar extent as the exposure of cells to the PPARδ ligand GW501516. Importantly, A-769662 significantly augmented PPARδ target gene expression in GW501516-treated cells. Similar results were obtained when analyzing mRNA expression of PPARδ target genes following macrophage treatment with GW501516 and salicylate, which has recently been recognized as another direct allosteric AMPK activator [37] (S1 Fig). Analysis of PPARδ target gene expression also revealed cell type-specific differences. Whereas the well-known PPARδ target gene angiopoietin-like 4 (Angptl4), which is a lipoprotein lipase inhibitor, was robustly induced in the THP-1 macrophage cell line, it was not induced in primary macrophages (S2 Fig).

To confirm the mRNA results of additively regulated genes affecting β-oxidation we performed Western analysis of FAO-associated targets PDK4 and CPT1a to test the effects of AMPK and/or PPARδ activation. Fig 2C shows that both PDK4 and CPT1a were elevated in response to GW501516, A-769662, or their combination as compared to untreated cells. However, the magnitude of the responses was less pronounced compared to mRNA expression changes. Assessment of FAO, measured as etomoxir-sensitive oxygen consumption in the presence of palmitate, revealed that although GW501516 induced moderate increases of FAO, A-769662 at concentration of 100 μM failed to do so (Fig 2D). Higher concentrations of A-769662 inhibited respiration (data not shown).

It has been shown that macrophage triglyceride (TG) accumulation induced by VLDL is significantly reduced by PPARδ activation [16]. To evaluate the effect of AMPK/PPARδ co-activation on VLDL-triggered foam cell formation, we treated primary macrophages with 100 nM GW501516 or 250 μM A-769662 for 48 hours and then stimulated cells with VLDL (20 μg/ml) for additional 24 hours (Fig 2E). VLDL-stimulation increased triglyceride accumulation in macrophages. Pre-treatment with A-769662 did not reduce the triglyceride amount, whereas the PPARδ agonist GW501516 significantly decreased triglycerides. No evidence for a stronger reduction in combined stimulation was observed.

**Fig 3:**
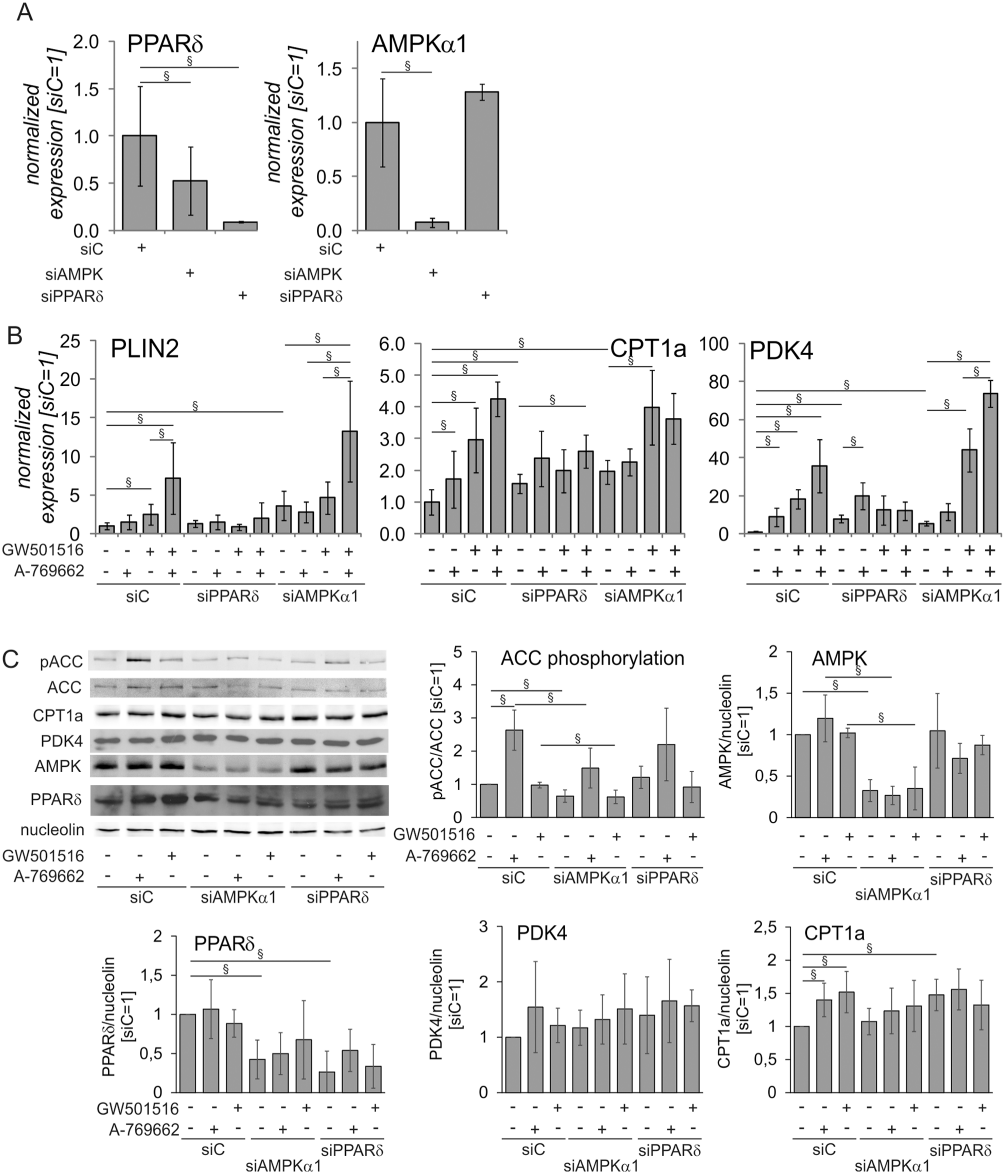
AMPK increased the expression of PPARδ target genes through PPARδ. Primary macrophages were transfected with 50 nM siControl (siC), AMPKα1 or PPARδ siRNA for 72 hours and treated with 250 μM A-769662 or 100 nM GW501516 for additional 24 hours. **A** mRNA expression of PPARδ and AMPKα1. **B** mRNA expression of PDK4, CPT1a, and PLIN2 in macrophages treated as indicated above. **C** Protein expression of phospho-ACC, ACC, PDK4, CPT1a, PPARδ, and AMPKα1 in macrophages treated as indicated above. Values represent averages ± 95% Confidence Interval. §, p<0.05 (n = 5–8).

To further dissect the roles of AMPK and PPARδ in regulating FAO-related gene expression, we silenced AMPK α1 catalytic subunit (the predominant isoform in human macrophages) and PPARδ and followed mRNA and protein expression of PPARδ target genes in macrophages treated with A-769662, GW501516 and their combination. Silencing of PPARδ achieved over 90% knockdown (KD) at the mRNA level (Fig 3A) and diminished the expression of PPARδ protein (Fig 3C). It also increased the basal expression of PPARδ target genes PDK4, CPT1a, but not PLIN2, consistent with the known repressor function of ligand-free PPARδ (Fig 3B) [38]. Cells with a PPARδ KD had also a blunted response to A-769662 and GW501516; PLIN2 mRNA expression was unaltered whereas PDK4 was significantly increased only in response to A-769662 and CPT1a was significantly increased only after co-treatment with A-769662 and GW501516. AMPKα1 KD achieved more than 90% reduction of AMPKα1 mRNA levels and over 65% reduction of AMPKα1 protein (Fig 3A and 3C). Accordingly, basal and A-769662-stimulated phosphorylation of the AMPK substrate ACC was significantly attenuated in AMPKα1-silenced cells (Fig 3C). Interestingly, AMPKα1 KD also reduced mRNA and protein levels of PPARδ and increased mRNA expression of PPARδ target genes thus, mimicking the PPARδ target gene mRNA expression changes in PPARδ KD cells (Fig 3A–3C). Similarly increased mRNA expression of PPARδ target genes after AMPKα1 knockdown was observed using unrelated siControl siRNA as well as in THP-1 macrophages stably transduced with unrelated AMPKα1 shRNA lentivirus (data not shown). AMPKα1 KD macrophages also did not show significantly increased mRNA expression of PPARδ target genes after A-769662 treatment. Still, AMPKα1 KD cells responded to PPARδ activation by GW501516 or combined GW501516/A-769662 treatment with increased expression of PLIN2, PDK4 or CPT1a. However, we did not observe any effect of A-769662 on PPARδ mRNA (data not shown). We also did not notice an effect of A-769662 treatment on nuclear PPARδ levels (S3 Fig).

Interestingly, Western analysis revealed only small changes of CPT1a or PDK4 protein expression under these experimental settings. Whereas we observed small, but significant increases of CPT1a protein after A-769 or GW501516-treatment in siControl-transfected macrophages, no differences were observed in AMPKα1 or PPARδ knockdown cells (Fig 3C), and siPPARδ-transfected cells also showed increased basal expression of CPT1a protein, reflecting, at least in part, mRNA data. For PDK4 similar tendencies were observed, but the changes did not reach statistical significance.

Collectively, these observations indicate that PPARδ mediates the effect of A-769662 and FAO-related target gene expression. Furthermore, AMPK may be involved in maintaining PPARδ expression through regulation of PPARδ mRNA. Surprisingly, changes in mRNA expression accompanying AMPKα or PPARδ knock-down are much less pronounced at the protein level for the PPARδ targets CPT1a and PDK4, indicating the discordance of mRNA and protein regulation of FAO genes in our experimental system.

## Discussion

Studies in mice have shown that simultaneous AMPK and PPARδ activation increased running endurance due to cooperative induction of oxidative and fatty acid metabolism in skeletal muscle [28]. As AMPK senses energy levels of the whole body we asked, if AMPK overexpression or activation can cooperate with the lipid metabolism regulator PPARδ and alter the expression of genes associated with fatty acid metabolism in human macrophages. Increased fatty acid catabolism might be beneficial under conditions of fatty acid oversupply associated with the metabolic syndrome [39]. Our results indicate that AMPK and PPARδ additively induce mRNA expression of a subset of fatty acid metabolism-related genes in primary human macrophages. We also show that the effect of AMPK on these genes is PPARδ-dependent. Despite robust changes of mRNA expression, the expression of proteins associated with FAO was only modestly enhanced by PPARδ/AMPK co-activation, and AMPK activation failed to further increase FAO or to prevent VLDL-induced TG accumulation in macrophages with activated PPARδ. Apparently, changes of the transcriptome alone may not be sufficient or are not pronounced enough to alter macrophage metabolic phenotype towards enhanced lipid catabolism.

For an unbiased approach to search for AMPK- and PPARδ-dependent genes, we performed microarray and quantitative PCR analyses of human primary macrophages. Cells were lentivirally transduced with a constitutively active construct coding for a truncated human AMPKα1 carrying mutation of Thr172 to an aspartic acid residue (T172D), treated with the PPARδ agonist GW501516, or the combination of both stimuli. Previous studies have shown that the T172D substitution in AMPKα resulted in about 50% increased activity of the kinase complex in comparison with the wild-type enzyme [40].

Our microarray analysis revealed FAO, pyruvate metabolism, TCA cycle, processes regulating kinesin binding, oxidative stress response, and ATP biosynthesis as the most affected pathways after combined PPARδ and AMPK activation. Addressing genes showing highest regulation in the FAO pathway we confirmed increased expression of PDK4, CPT1a, and PLIN2 mRNAs. Similar results were obtained when overexpressing the regulatory AMPKγ1 subunit with activating R70Q mutation. Furthermore, we observed that the same transcripts were up-regulated after pharmacological activation of AMPK using the direct allosteric activators A-769662 or salicylate [26, 37]. PDK4 is a mitochondrial kinase, which phosphorylates and inactivates pyruvate dehydrogenase, inhibiting the formation of glucose-derived acetyl-CoA. In tissues with high metabolic flexibility, such as skeletal muscle, increased PDK4 expression is a major regulatory switch, reducing glucose oxidation in favor of increased β-oxidation [41, 42]. CPT1a is a rate-limiting enzyme of FAO transferring long-chain acyl-CoA into mitochondria and serves as a key regulatory enzyme of β-oxidation [39]. In addition, PLIN2, a typical PPARδ target protein associated with cytosolic lipid droplet stabilization [43], was additively induced by AMPK and PPARδ.

Although we observed robustly increased mRNA levels of PDK4 and CPT1a (Figs 1B and 2B), only small differences appeared at the protein level (Figs 2C and 3C). This may reflect the known ability of AMPK to generally suppress protein synthesis by interfering with the mTOR pathway, which we confirmed in our system (Fig 2A) [8]. Furthermore, while PPARδ activation induced a moderate increase of FAO, this was not seen with AMPK activation (Fig 2D). Similarly, suppressing triglyceride accumulation by GW501516 is not significantly enhanced by AMPK activation (Fig 2E). Apparently, the small increase of CPT1a and PDK4 protein expression observed after combined AMPK/PPARδ activation does not translate to the enhancement of FAO or triglyceride lowering achieved by activation of PPARδ alone. It should be noted that FAO is governed not only by amounts of the corresponding enzymes or substrates, but also to a major extent by the ATP demand. This may explain our observations that dramatic increases of CPT1a protein expression were accompanied by only a 2-fold increase in FAO in CPT1a-overexpressing THP-1 macrophages [39]. Since the major outcome of pharmacological AMPK activation in the absence of falling ATP levels is the suppression of ATP-consuming processes, this may decrease ATP demand and negatively affect ATP generation, including that provided by FAO. The complexity of FAO regulation has been recently illustrated by a study of mutated ACC double knock-in mice where changes of a major allosteric FAO modulator malonyl-CoA had no impact on cardiac FAO rate [44]. The insensitivity of FAO to malonyl-CoA levels may also apply to our system. While observing robust changes of phospho-ACC after AMPK activation, we fail to observe a significant impact on FAO. It can be envisioned that strategies aiming at increasing ATP demand, such as increasing energy dissipation through mitochondrial uncoupling [45], may be needed to translate enhanced expression of fatty acid catabolic genes into increased FAO in this setting. Mechanistically, suppression of VLDL-induced TG accumulation in macrophages treated with PPARδ ligands was attributed to both, enhanced FAO and increased expression of the lipoprotein lipase inhibitor Angptl4 [16, 46]. Although we observed a robust induction of Angptl4 mRNA by GW501516 in THP-1 cells, the same PPARδ ligand failed to induce Angptl4 in primary human macrophages (S2 Fig), indicating that FAO is the primary pathway for TG reduction in the human system.

FAO has been suggested to promote an anti-inflammatory phenotype of macrophages polarized by IL-4 [47, 48]. Both PPARδ and AMPK can contribute to anti-inflammatory macrophage polarization [49, 50], however whether increased FAO is critical for anti-inflammatory effects of activated PPARδ or AMPK is unclear. Our recent observations indicate that FAO is dispensable for IL-4-induced human macrophage polarization [51], suggesting important differences between human and mouse macrophages regarding the impact of metabolism on macrophage phenotype. Thus, FAO may play more important roles to specifically attenuate saturated fatty acid-induced inflammation [25, 39].

Previous data in skeletal muscle suggested that AMPK may directly activate PPARδ transcriptional activity [28], although mechanistic details remain obscure. Our approach to silence PPARδ confirmed that the increased mRNA expression of PPARδ target genes in cells co-activated with AMPK is indeed PPARδ-mediated (Fig 3). Consistent with previous observations PDK4, CPT1a, and PLIN2 are actively repressed by ligand-free PPARδ in human macrophages, reflected by their mRNA increase in PPARδ-depleted cells [38, 52, 53]. Accordingly, AMPK or PPARδ activation does not influence the expression of these genes after a KD of PPARδ, indicating an AMPK impact on FAO-associated genes through PPARδ. In accordance to our data, recent study showed that reduction of ER stress in vascular cells by AMPK activator metformin was dependent on PPARδ activity [54]. Interestingly, AMPKα1 KD data revealed a role for AMPK in control of PPARδ mRNA and protein expression, although AMPK activation did not affect PPARδ mRNA, suggesting that the impact of AMPK on PPARδ expression may be independent of AMPK catalytic activity. However, we do not see the reduction of PPARδ target gene induction by GW501516 in AMPKα1 knockdown cells, and we noticed no activation of AMPK substrate phosphorylation by GW501516, indicating that AMPK is not downstream of PPARδ in our system. Although AMPK-mediated reduction of ER stress in muscle cells by GW501516 has been recently reported [55], this study used high concentration of GW501516 (10 μM), which changed the cellular AMP/ATP ratio. We also noticed that expression of some PPARδ target genes, such as ACAA2, ACADVL or FABP4, was not affected by AMPK activation (data not shown), suggesting that modulation of PPARδ activity may be gene-specific and not through direct PPARδ activation. Accordingly, no changes of PPARδ nuclear levels were noticed by us. Similarly, no evidence of direct post-translational modification of PPARδ by AMPK was found in a previous study investigating the interaction of these proteins in muscle cells [28]. Instead, several alternative mechanisms linking AMPK to altered genes expression may be envisioned, such as modification of histone deacetylases [56].

In summary, our data indicate that activated AMPK increases PPARδ-dependent expression of a subset of genes involved in fatty acid metabolism, which requires the transcriptional activity of PPARδ. Potentiation of gene expression on its own is unable to increase FAO and prevent VLDL-induced lipid accumulation, suggesting that additional interventions to increase fatty acid catabolism are needed to therapeutically exploit AMPK/PPARδ interaction in the macrophages.

## Supporting Information

S1 Fig Effects of salicylate on mRNA expression of PPARδ target genes. Primary macrophages were stimulated by 100 nM GW501516 and 3 mM salicylate for 24 hours. mRNA expression was analyzed by quantitative PCR. Values represent averages ± 95% Confidence Interval. *, p<0.05 (n = 5).
(TIF)

S2 Fig Gene expression of Angptl4 in human macrophages. THP-1 (**A**) or primary macrophages (**B**) were stimulated by 100 nM GW501516 for 24 hours. mRNA expression of Angptl4 was analyzed by quantitative PCR. Values represent averages ± 95% Confidence Interval. *, p<0.05 (n = 4).
(TIF)

S3 Fig Effect of A-769662 on nuclear PPARδ levels. Western blotting of PPARδ in nuclear extracts of primary macrophages stimulated with 500 μM A-769662 for 24 hours. Values represent averages ± 95% Confidence Interval.
(TIF)
